# Feeding Problems and Long-Term Outcomes in Preterm Infants—A Systematic Approach to Evaluation and Management

**DOI:** 10.3390/children8121158

**Published:** 2021-12-08

**Authors:** Ranjith Kamity, Prasanna K. Kapavarapu, Amit Chandel

**Affiliations:** 1 Division of Neonatology, Department of Pediatrics, NYU Langone Hospital-Long Island, NYU Long Island School of Medicine, Mineola, NY 11501, USA; 2 Section of Neurogastroenterology & Motility, Division of Pediatric Gastroenterology, Hepatology & Nutrition, Children’s Hospital of Philadelphia, Perelman School of Medicine at the University of Pennsylvania, Philadelphia, PA 19104, USA; kapavarapp@chop.edu; 3 Division of Neonatology, Department of Pediatrics, Atrium Health Wake Forest Baptist, Winston-Salem, NC 27157, USA; achandel@wakehealth.edu

**Keywords:** prematurity, outcomes, dysphagia, oral feeding, deglutition, videofluoroscopic swallow study (VFSS), fiberoptic endoscopic evaluation of swallowing (FEES), manometry, feeding problems

## Abstract

Preterm infants are known to have long-term healthcare needs. With advances in neonatal medical care, younger and more preterm infants are surviving, placing a subset of the general population at risk of long-term healthcare needs. Oral feeding problems in this population often play a substantial yet under-appreciated role. Oral feeding competency in preterm infants is deemed an essential requirement for hospital discharge. Despite achieving discharge readiness, feeding problems persist into childhood and can have a residual impact into adulthood. The early diagnosis and management of feeding problems are essential requisites to mitigate any potential long-term challenges in preterm-born adults. This review provides an overview of the physiology of swallowing and oral feeding skills, disruptions to oral feeding in preterm infants, the outcomes of preterm infants with feeding problems, and an algorithmic approach to the evaluation and management of neonatal feeding problems.

## 1. Introduction

Occurring in over 10% of live births in the world annually, prematurity is considered a significant global healthcare problem [1,2]. In the USA, preterm births account for about 400,000 births per year, and their outcomes will define the health of a significant proportion of the next generation [1]. Preterm infants face several challenges during their neonatal intensive care unit (NICU) stay, including cardiorespiratory problems, infections, and neurological challenges, while continuing to grow and attain neurodevelopmental milestones for NICU discharge [3]. The respiratory morbidities and neurodevelopmental delays related to prematurity pose a heavy burden on the patients, families, and the healthcare system [4].

Insults during organogenesis and tissue differentiation can impact the developing fetus or preterm infant, resulting in short-term and long-term implications. Developmental plasticity helps the fetus develop a phenotype best suited to the environment, but these short-term adaptations could result in the future development of pathological states [5]. Barker et al. hypothesized that malnutrition during gestation could alter fetal metabolic programming leading to early-onset heart disease as adults [6]. Prenatal factors such as maternal malnutrition, disease, environmental exposures, maternal stress, and lifestyle can affect the developmental programming of the fetus by altering the maternal–fetal endocrine axis, leading to placental abnormalities, fetal growth restriction, and compensatory postnatal growth [7,8].

Oral feeding readiness in preterm infants is a concern often towards the tail-end of hospitalization. This only comes into the spotlight after other major medical concerns have been resolved or are manageable. While the evidence for the adult outcomes of preterm infants is increasing, the long-term impact of the altered development of oral neuromotor skills and abnormal oral feeding remains unclear, primarily due to a paucity of studies on adults born preterm. Given the diversity of underlying etiologies and clinical presentations of feeding problems, the lack of standardized definitions and assessments by which to identify and document oral feeding problems adequately is frustrating [9]. There is an urgent need for a two-pronged approach to feeding problems in infants born preterm in order to standardize diagnostic criteria and to develop individualized therapeutic strategies using a comprehensive multidisciplinary approach. 

In this review, we will appraise (1) the physiology of swallowing and oral feeding skills, (2) disruptions to oral feeding in preterm infants, (3) feeding problems in preterm infants beyond the NICU, and (4) an algorithmic approach to the evaluation and management of neonatal feeding problems. 

## 2. Understanding Oral Feeding and Feeding Problems in Preterm Infants

Normal swallowing begins with the formation of a food bolus in the mouth and ends with the bolus being carried down into the stomach through a series of coordinated functions involving sucking, swallowing, respiratory endurance, and airway protection with overarching neurological control. Anatomical changes such as decreasing relative tongue size and lower laryngeal position with growth play an important role in the evolution of the swallowing mechanisms [10]. 

### 2.1. Development of Normal Swallowing in the Fetus

The precursors to oral feeding skills emerge in the early second trimester of pregnancy with evidence of sucking and swallowing in the developing fetus. Early evidence of fetal swallowing is seen at 11–12 weeks of gestation by the swallowing of amniotic fluid, followed by evidence of early sucking at 18–20 weeks [10,11]. As the fetus enters the third trimester, the sucking and prefeeding skills are further refined, resulting in a mature sucking by term gestation. In an infant born preterm, the mature sucking–swallowing coordination is preceded by non-nutritive sucking (NNS) at around 28–33 weeks, which refers to repetitive sucking on a pacifier or a closed nipple without resulting in the delivery of a liquid bolus for swallowing [12,13]. NNS occurs in short rapid bursts, which slows down as the sucking matures into a 1Hz sucking–swallowing–breathing pattern of nutritive sucking (NS) by week 32–34 of gestation with improved rhythmicity, amplitude, and volume expression [11,13,14]. 

Mature swallowing occurs broadly in three phases—the oral phase, the pharyngeal phase, and the esophageal phase [15,16]. The oral phase in infancy is synonymous with the sucking that creates a vacuum followed by the expression of milk by the tongue compressing on the bottle or breast. Normal sucking is tied to receiving the appropriate sensory input from food, followed by a coordinated oral motor response. In the neonatal period, this is thought to be regulated by bilateral brainstem neural networks in the pontine and medullary reticular formation, called the suck central pattern generator (CPG). The CPG is responsive to stimulation and can be trained using oral stimulation with pacifiers, nipples, breastfeeding, and olfactory, tactile, or thermal stimulation [13,17,18,19]. Sucking is reflexive in neonates and becomes more voluntary as the infant grows and learns to masticate [15].

As the food bolus is pushed to the back of the oral cavity, a largely involuntary swallowing reflex is triggered in the pharyngeal phase. This leads to a sequence of events from the elevation of the velum leading to nasopharyngeal closure, followed by the pharyngeal swallowing reflex with the contraction of superior, middle, and inferior constrictors (resulting in pharyngeal closure). A brief respiratory pause with the closure of the vocal cords from the glottic closure reflex and laryngeal chemoreflexes help protect the airway during this phase. As the bolus moves to the esophageal phase, this activates a series of actions from hyoid elevation, relaxation of the cricopharyngeal muscle, opening of the esophagus, and the eventual peristaltic contraction of the esophagus (upper esophageal sphincter (UES) relaxation, primary peristalsis of the esophageal body, and lower esophageal sphincter (LES) relaxation) that moves the food bolus into the stomach, thus concluding the swallow [10].

### 2.2. Feeding Problems in Preterm Infants in the Neonatal Period

The phrase “feeding problems” is often used to describe a diverse group of etiologies resulting in inadequate nutritional intake orally and could range from oral feeding difficulties (described in more detail below), gastroesophageal reflux (GER), dysmotility, and even systemic illnesses, unfortunately contributing to the lack of a uniform definition for feeding problems. The maturation of a normal feeding pattern can be interrupted in a preterm infant by extrauterine influences resulting in dysphagia or abnormal swallowing, with impaired sucking, swallowing, or airway protection, thus compromising the efficacy, adequacy, and, most importantly, the safety of oral feeding. Dysphagia can be a sign of underlying anatomical or neurological abnormality or injury, or merely functional immaturity altered by external sensory input, as seen in a preterm infant [16]. Infants with dysphagia are phenotypically heterogeneous and have diverse etiologies, pathophysiology, and management. 

**Figure 1:**
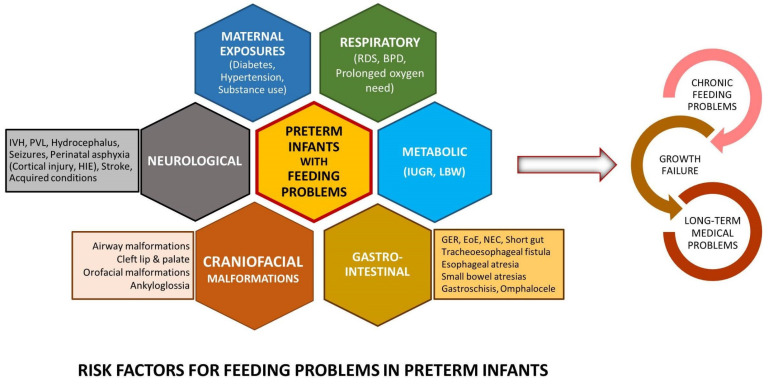
Risk factors: Common clinical conditions associated with feeding problems in preterm infants. (BPD: bronchopulmonary dysplasia, EoE: eosinophilic esophagitis, GER: gastroesophageal reflux, HIE: hypoxic ischemic encephalopathy, IUGR: intrauterine growth restriction, IVH: intraventricular hemorrhage, LBW: low birth weight, NEC: necrotizing enterocolitis, PVL: periventricular leukomalacia, RDS: respiratory distress syndrome).

Preterm infants, especially those born <32 weeks, are exposed to multiple life-saving interventions during their NICU stay that contribute to noxious orofacial sensory stimulation during a time of physiologic maturation. Additionally, during such a vital period of brain development, prolonged oxygen supplementation due to respiratory illnesses (respiratory distress syndrome (RDS) and bronchopulmonary dysplasia (BPD)), brain injury (intraventricular hemorrhage (IVH), periventricular leukomalacia (PVL) or hypoxic injury), complex medical illnesses such as necrotizing enterocolitis (NEC), surgeries, and underlying craniofacial abnormalities can further alter oral sensory and motor experiences impacting normal sucking and swallowing development. There exists a complex interplay between prematurity, pulmonary, neurological, and feeding problems independently linked to neurodevelopmental delays. Figure 1 shows a selected list of common conditions linked to dysphagia. Dysphagia can also be mechanistically categorized by phases of swallowing with overlapping mechanisms. 

Oral phase disruptions: The oral phase is altered in preterm infants who experience prolonged external stimulation (oral or nasal endotracheal tubes, non-invasive respiratory support interfaces, orogastric or nasogastric tubes, tapes, and securing devices). Immature or absent oral reflexes result in subtle to overt symptoms from an abnormal oral phase, including weak and disorganized sucking, immature patterns such as biting or chewing, and poor bolus formation and propulsion, leading to dribbling during feeding [15,16]. Breastfeeding failure is also a frequent concern. 

Pharyngeal phase disruptions: The swallowing mechanism is intricately linked to respiratory efficiency. The pharynx is the only common pathway between the airway and digestive tract during swallowing and requires a coordinated response between swallowing and breathing for successful feeding [20]. Immature sucking–swallowing–breathing coordination during feeding due to an abnormal or absent swallow reflex, or delayed swallow initiation, can compromise airway safety resulting in food particles entering the nasopharynx (nasopharyngeal reflux) or the airway, leading to laryngeal penetration (food particles entering the larynx but above the vocal cords) or tracheal aspiration (food particles entering the trachea). Sucking–swallowing–breathing incoordination can present with choking, gagging, apneas, bradycardia, or desaturations during feeding [21].

Esophageal phase disruptions: The pharyngeal reflexive swallow and pharyngo-lower esophageal sphincter reflex were previously shown by Jadcherla and colleagues to be immature and underdeveloped in preterm infants compared to term infants [21]. Additionally, the presence of retrograde esophageal peristalsis and non-peristaltic esophageal motility in preterm infants contributes to abnormal esophageal clearance in preterm infants [10,16]. Immaturity or compromise of the esophageal swallow reflexes can result in the food bolus refluxing upwards in the esophagus up to the airway (reflux (GER). The refluxed acidic gastric contents can irritate the esophageal mucosa and cause painful swallowing (odynophagia), which is often a precursor for feeding aversion. The esophageal mucosal bicarbonate secretion acts as an additional protection against GER to some extent, but can be overcome by the quantity and severity of GER. 

**Figure 2:**
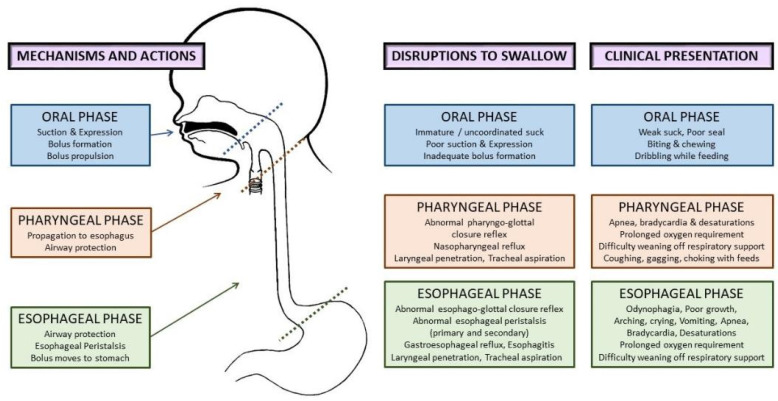
Etiopathophysiology of dysphagia in preterm infants: phases of normal swallowing, disruptions to swallowing, and clinical presentation in the neonatal period.

Aspiration during swallowing can occur broadly in three scenarios—anterograde aspiration in the oropharyngeal phase as bolus travels into the pharynx, retrograde aspiration (refluxed food bolus travels up the esophagus with failure to protect the airway), and silent aspiration (asymptomatic aspiration anytime during swallowing). Additionally, an abnormal pharyngoglottal closure reflex and esophagoglottal reflex associated with prematurity also contribute to abnormal airway protective mechanisms. Figure 2 shows the phases of swallowing, normal actions in each phase, disruptions to swallowing, and their clinical presentation. In the neonatal period, feeding problems can present with diverse clinical manifestations, as shown below, leading to inadequate nutritional intake, increased length of hospitalization, and compromised airway safety [9,10,16].

Oral feeding and non-invasive respiratory support: It is not uncommon for preterm infants who are starting to develop oral feeding skills at 33–34 weeks postmenstrual age to continue to require respiratory support due to lung disease (RDS or BPD). Oral feeding such preterm infants on moderate respiratory support such as nasal CPAP or high-flow nasal canula (HFNC) has been debated over the years. Some studies have shown that oral feeding on CPAP may be associated with increased risk of aspiration [22,23], while others showed that feedings were tolerated safely on non-invasive support [24]. Some centers continue to feed on CPAP or HFNC to promote oral motor stimulation in hopes of shortening the hospital length of stay. In a recent retrospective pre–post study regarding feeding infants while on CPAP and not feeding on CPAP, the latter group achieved full oral feeds at the same postmenstrual age as the first group, suggesting that earlier attempts at feeding may result in prolonged ineffective feeding with the same end result [25]. Despite safety concerns of oral feeding while on CPAP, the clinical significance and long-term impact have been debated [26,27].

## 3. Feeding Problems in Preterm Infants beyond the NICU

There is currently a dearth of large-scale studies describing feeding problems in children and adults born preterm, and the descriptions of feeding problems vary significantly between previous studies with a high degree of heterogeneity. In a large NICHD neonatal research network (NRN) study, Adams-Chapman et al. defined abnormal feeding as the need for gastrostomy or tube feeds, choking, gagging, excessive drooling, or difficulty swallowing during oral feeds, physicians’ orders to not feed by mouth, and confirmed aspiration [28]. Conversely, another study used the need for thickened feeds as a criterion, without clear criteria for the use of thickened feeds [29].

### 3.1. Prevalence and Extent of Oral Feeding-Related Adverse Outcomes

Feeding problems in childhood and adults among preterm born infants have been described inconsistently. While previous studies have suggested that up to 1% of the pediatric population can present with feeding problems, the prevalence is likely to be significantly higher in preterm infants, proportional to the degree of prematurity [15]. Residual feeding problems in growing preterm infants span a broad spectrum of phenotypes, including feeding tube dependency [30], non-specific feeding difficulties, oral aversion [9,31,32], feeding aversion, poor progression to solid foods, challenges with eating specific food types (e.g., solids), and subtle swallowing difficulties. 

As the feeding skills mature into childhood with more voluntary control, it is only plausible that feeding problems become more apparent in infants with neurological injury and impaired cortical function. In a recently published meta-analysis by Pados et al., including 22 studies, the prevalence of feeding problems was 42% in the first four years of life amongst preterm-born infants [9]. In a subgroup of 11 studies including almost 2500 extreme preterm infants <28 weeks, the prevalence of feeding problems was 46%, with significant variation among studies. At 28–32 weeks of gestation, i.e., very preterm infants, nine studies showed significant differences amongst groups with an overall prevalence of 42%. Further breakdown by age showed that the prevalence decreased slightly with advancing age, from 43% at 0–5 months, down to 33% at 24–48 months [9]. It is critical to note that the authors in this study reported the apparent lack of uniform definitions of feeding problems as a major limitation. 

In a retrospective cohort of 194 preterm infants of <37 weeks with feeding difficulties referred to a specialized feeding disorders program, 40% of infants were discharged on gastrostomy tube (GT) feeds, and out of these, the majority (78%) were still receiving GT feeds at one year of age, either full GT (40%) or partial GT feeds (38%) [30]. These GT numbers are higher than those in the general preterm population, as this study only included a subset of preterm infants with feeding difficulties who were referred for evaluation for a GT and would be expected to have feeding tube dependency. In another large NICHD study in extremely low birth weight (ELBW) infants including 25 centers, 333 (7.3%) out of 4549 ELBW infants required GT placement [33]. In a prospective study conducted in Austria as part of the initiative to wean tube feeds, out of the 711 children enrolled in the cohort, 53% were preterm, and a majority (two thirds) of the preterm infants were on tube feeds since birth. The authors also reported that amongst the extreme preterm infants, 83.5% had no other diagnosis except prematurity and its complications, pointing towards a strong association between feeding tube dependency and prematurity [34,35].

Postnatal malnutrition associated with feeding problems can result in failure to thrive (FTT) and developmental delays. Postnatal nutritional compromise can modify adult outcomes in these children resulting in multisystem abnormalities as an adult. In the NICHD study mentioned above, Warren et al. also showed a high incidence of FTT amongst the infants discharged home on GT feeds. Out of the 333 ELBW infants (7.3%) that were discharged on GT, 56% had FTT at an 18–22-month follow-up [33]. In the short term, most notably, the pulmonary consequences resulting from impaired airway protection can damage the already delicate lungs leading to worsened BPD, prolonged oxygen requirement, ventilator support, and longer length of hospitalization. Infants with prolonged intubation or tracheostomy could have dysphagia due to incompetence of the palatopharyngeal complex, impaired subglottic pressure, and laryngeal elevation [36,37,38,39]. Improving pulmonary function into childhood and adulthood is expected to improve overall pulmonary compliance resulting in improvement in a subset of feeding problems related to respiratory endurance. While this may partially be true, especially in the milder cases, there is a lack of evidence supporting or refuting the claim at this time.

Oral aversion was described in a population survey conducted in the UK at two years corrected age comparing 1130 preterm versus 1255 term infants. The authors identified an increased incidence of feeding difficulties, picky eating/refusal, and oral motor problems in the preterm infant cohort compared to those born term. The adjusted relative risk for total feeding problems was 1.57, refusal/picky eating was 1.53, and oral motor problems was 1.62. According to a multivariate analysis, the authors noted that prolonged nasogastric tube >2 weeks was independently associated with increased eating difficulties [32]. Speech and language delays are closely associated with feeding problems as the neural pathways for feeding and speech are inextricably linked; these infants often present with speech-language delays. Adams-Chapman et al. described that infants with a history of feeding problems have a higher incidence of language delays in early childhood [40]. Early referral to speech therapy is indicated in these infants.

Gastrointestinal reflux is a frequent indication for the use of thickened feeds and anti-acid medications in preterm infants. A population based study based on the Swedish birth registry showed an inverse relation between gestational age and the use of anti-acid medications per year for gastroesophageal reflux disease in adulthood [41]. Of all the adults aged 25–37 years age, 5.5% of those born at 22–27 weeks gestational age were on at least one anti-acid medication compared to 1.7% of those born at term (37–42 weeks). The adjusted odds ratio for being on at least on one anti-acid medication per year was 3.38 for those born at 22–27 weeks, 1.38 for 28–34 weeks, and 1.19 for 35–37 weeks compared to those born at term (37–42 weeks gestational age) [41].

Eosinophilic esophagitis (EoE) is chronic immune-mediated inflammatory disease of the esophagus characterized by eosinophilic inflammation of the esophagus. Multiple case–control studies in both pediatrics and adults have shown that prematurity is one of the early-life risk factors for developing eosinophilic esophagitis in one’s later years of life. In a case–control study comparing children with EoE versus healthy controls, premature infants had 46% higher odds of developing EoE compared to term infants, with an adjusted odds ratio of 1.46. Multiple pediatric and adult studies have shown a variable odds ratio of 1.46 to 2.92 for prematurity being an early-life risk factor for developing EoE in childhood or adulthood [42,43,44].

### 3.2. Feeding Problems in Special Populations

Feeding problems are described in multiple genetic syndromes associated with hypotonia in infancy. Term infants with trisomy 21, even in the absence of major anomalies (40%), have more feeding problems when compared to their term counterparts (34% vs. 15%) without trisomy 21 [45]. Infants with Prader–Willi syndrome can present with hypotonia and poor sucking even in the neonatal period and have feeding problems and failure to thrive [46,47]. Additionally, congenital deformities of the face and oropharyngeal structures are a strong predictor of feeding problems. Some may be readily apparent (e.g., cleft lip and palate, Robin sequence), while others are less obvious, requiring a careful physical exam. Syndromes such as Coffin–Siris syndrome with distinctive craniofacial abnormalities and hypotonia may cause feeding difficulty even in the absence of any other gastrointestinal issues [48]. Infants with surgical conditions such as gastroschisis and repaired esophageal atresia are also at increased risk of oral feeding problems from prolonged illness, delayed initiation of oral feeding, and associated complex medical illnesses. 

Congenital heart diseases have long been linked to feeding problems even after several years following cardiac surgery, leading to a substantial number of infants needing nasogastric tubes or GT for nutrition [49,50]. Feeding problems in this group are often a result of poor cardiorespiratory endurance, delayed initiation and transition to oral feeds, and other medical complications related to underlying medical issues [51]. Prolonged intubation and an increased risk-adjusted congenital heart surgery score seemed to be predictive of feeding problems [50]. A common microdeletion syndrome involving chromosome 22 q11.2 affects multiple systems and manifests as congenital heart disease with severe feeding difficulties partly due to velopharyngeal incompetence and palatal abnormalities [52].

Maternal metabolic conditions such as hypertension, diabetes, and drug use have been linked to feeding problems in neonates. Infants of diabetic mothers have an increased prevalence of feeding issues during the early neonatal period. There is increasing evidence that feeding and eating disorders persist in infancy and early childhood. A population-based cohort study from Denmark showed a 64% increased risk of feeding and eating disorders in children of mothers with diabetes [53]. Neonates exposed to opioids prenatally are a unique subgroup reported to have poor feeding efficiency, nipple rejection, hiccups, spitting, coughing, prolonged sucking bursts with fewer pauses, irregular sucking rhythm, and increased arousal [54,55]. These infants can be hyperphagic initially and are likely to have uncoordinated sucking–swallowing–breathing, placing them at risk for aspiration [54].

### 3.3. Outcomes in Preterm Infants with Potential Link to Feeding Problems

Intrauterine growth restriction (IUGR) is linked to nutritional compromise in a fetus, preventing it from reaching its full genetic potential, and it is principally a vascular disorder with short-term and long-term consequences [56,57,58]. There is a considerable void in the data on feeding problems in this cohort of preterm, low birth weight infants and their long-term outcomes. Feeding problems in these infants could signal functional immaturity of the central nervous system but can also be a sign of neurological injury or adaptability to being born preterm. Feeding problems impact more than nutrition in children and adults born preterm, often presenting with subtle difficulties. Specific signs are usually absent, and the subtlety of these minor signs makes it more challenging to identify and intervene timely and to potentially offer a chance to improve outcomes.

Preterm infants are also prone to higher rates of anoxic brain injury and IVH, with increased incidence of cerebral palsy often manifesting as subtle dysphagia. While the data on the long-term outcomes of preterm infants discharged home on tube feedings are sparse, term infants with congenital cardiac disease who are discharged home on an artificial mode of providing nutrition such as orogastric tubes or GT have poorer mental and psychomotor developmental indices [59]. It would suffice to hypothesize that infants born preterm will more than likely have at least similar or worse neurodevelopmental issues when compared to their term peers.

## 4. Addressing Oral Feeding Issues Early

**Figure 3:**
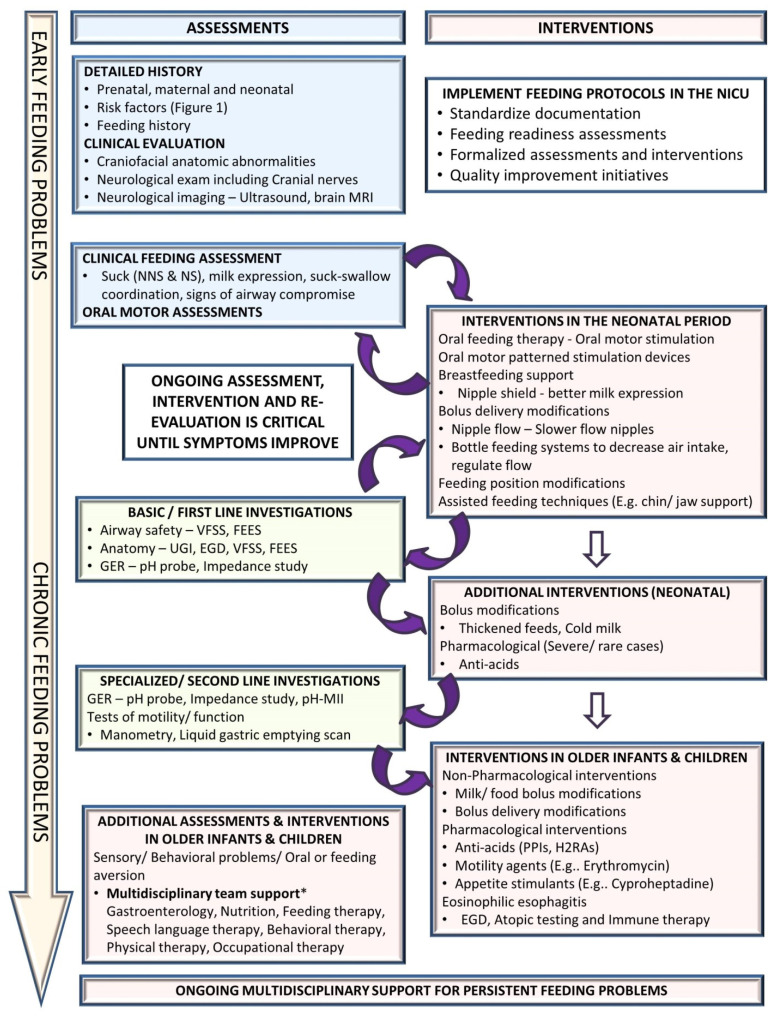
Approach to a preterm infant with feeding problems. (EGD: esophagogastroduodenoscopy, FEES: fiberoptic endoscopic evaluation of swallowing, GER: gastroesophageal reflux, H2RA: H2-receptor antagonists, MRI: magnetic resonance imaging, NICU: neonatal intensive care unit, NNS: non-nutritive suck, NS: nutritive suck, pH-MII: pH monitoring multichannel impedance, PPIs: proton pump inhibitors, UGI: upper gastrointestinal fluoroscopy, VFSS: videofluoroscopic swallow study).

Feeding problems are dynamic and continue to evolve as the infant grows, requiring an ongoing assessment, intervention, and re-evaluation to optimize management. Early diagnosis and treatment of feeding problems involve a holistic approach to the individual’s specific feeding difficulties in order to decrease potential long-term challenges [16,19]. Figure 3 suggests an algorithmic approach for managing feeding problems in the neonatal period continuing into childhood. 

### 4.1. Clinical Assessment

A detailed history includes prenatal history, maternal medical problems and exposures, birth history, neonatal course by systems, and a description of the signs and symptoms. Physical examination should evaluate the anatomy of the oropharyngeal structures and identify craniofacial anomalies and neurological status, including cranial nerves. The quality and maturity of the sucking, milk expression, and sucking–swallowing coordination are assessed. Abnormalities during a bedside feeding, such as wet voice, gagging, choking, and drooling, could suggest a possible airway compromise that may require targeted instrumental assessment. However, it is essential to note that the pediatric population is suspected of having a high prevalence of silent aspiration of unknown significance and may not be identified accurately on clinical assessments alone. 

Several bedside tools are now available for oral motor assessment of infant feeding skills. Some commonly used tools in the neonatal period include Infant Driven Feeding Scales (IDFS), Neonatal Oral Motor Assessment Scale (NOMAS), the Oral Feeding Skills Scale (OFS), and newer tools such as the Neonatal Eating Assessment Tool (NeoEAT) and Neonatal Feeding Assessment Scale (NFAS) [60,61,62,63]. Other scales used for evaluating pediatric dysphagia include Schedule for Oro-Motor Assessment (SOMA), Montreal Children’s Hospital Feeding Scale, and the Dysphagia Disorder Survey. Numerous advanced speech and language assessment tools are also available for a detailed evaluation of specific speech-language concerns. However, informal clinical assessments are used more frequently in the NICU than formal assessment tools [9].

### 4.2. Non-Pharmacological Interventions

Multidisciplinary feeding teams including neonatology, speech pathology, and occupational and physical therapy are integral to understanding the intricacies of feeding issues related to prematurity and the complex interplay of other comorbidities. Neonatal interventions for dysphagia are primarily conservative, involving non-pharmacological management. Feeding readiness assessments for maturity and respiratory safety—informal or structured—are essential to guide the initiation of oral feeds in a preterm infant. Oral motor therapy is an essential element for the improvement of readiness to start feeding [11,17]. The pace of transition from tube feeds to oral feeds varies by the level of maturity of the infant. Feeding modifications using changes in feeding position, chin, cheek, or jaw support, and changes in nipple flow rates are frequently used in the neonatal period [15,64,65]. Breastfeeding support using nipple shields can aid in improved milk expression. As the infants mature, persistent feeding problems may require instrumental assessments, as mentioned in the next section. 

Bolus modifications such as thickened feeds may be used in some cases as the infant becomes closer to term gestation [66,67]. In a retrospective cohort of 137 children (including 35% preterm infants) under two years of age who had laryngeal penetration on a videofluoroscopic swallow study (VFSS), significant improvement was noted amongst children who received thickened feeds (91%) compared to those who had change in flow rate (36%) and those who received no interventions (19%) [66]. Other novel feeding modifications such as cold liquid feedings have been shown to decrease the risk of aspiration in a small pilot study, but these are not yet used widely in clinical practice [68]. As infants approach term gestation, signs of GER with associated apnea, bradycardia, or desaturations often delay NICU discharge. Thickening and feeding modifications have been used as first-line therapy in addition to a trial of an extensively hydrolyzed amino acid-based formula [69]. During this phase, optimizing nutrition, including possible breastfeeding or breastmilk use, remains essential for adequate growth, which is turn helps improve developmental outcomes and other comorbidities such as BPD.

### 4.3. Instrumental Assessment

**Figure 4:**
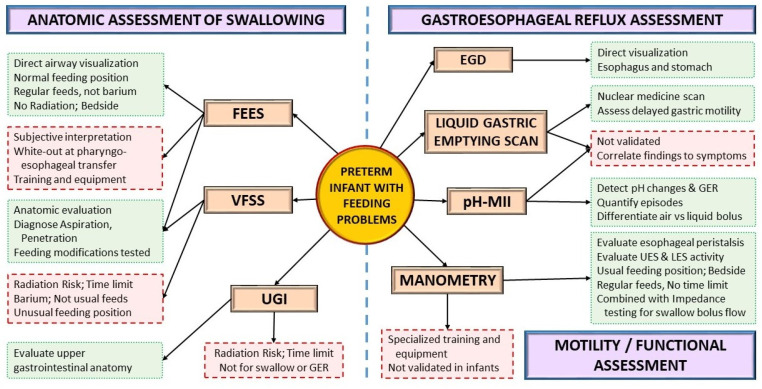
Instrumental assessment for feeding problems. Green boxes with dotted outlines depict advantages; red boxes with dashed outlines depict disadvantages. (EGD: esophagogastroduodenoscopy, FEES: fiberoptic endoscopic evaluation of swallowing, GER: gastroesophageal reflux, LES: lower esophageal sphincter, pH-MII: pH monitoring multichannel impedance, UES: upper esophageal sphincter, VFSS: videofluoroscopic swallow study, UGI: Upper Gastrointestinal Fluoroscopy).

Instrumental assessments can be used to evaluate the anatomy or function of aerodigestive structures and the presence of GER as the infant grows (Figure 4). A VFSS or modified barium swallow study provides a fluoroscopic view of the swallow while the infant is fed barium at different consistencies. VFSS is one of the most widely used instrumental assessments for dysphagia in neonates and infants [70,71]. Conversely, fiberoptic evaluation of swallowing (FEES) provides a direct view of the airway during actual feeding, without the risk of ionizing radiation, but with a momentary white-out period at the pharyngo-esophageal transfer. Introduced in the 1980, FEES has only recently gained popularity in the neonatal population due to the development of smaller neonatal-sized endoscopes [72,73,74,75,76,77,78]. 

Both VFSS and FEES are complementary studies that can provide different perspectives. Studies comparing VFSS and FEES showed significant agreement between the two [75,76]. An upper gastrointestinal fluoroscopic (UGI) study can also identify anatomical abnormalities of the esophagus and upper small bowel, including malrotation, gastric outlet obstruction, abnormal esophageal clearance, hiatal hernia, and abnormal duodenal clearance. In a prospective study conducted in infants less than one-year-old, the GER noted on the UGI did not correlate with pH testing, highlighting the need to interpret reflux findings on UGI cautiously [79]. 

pH-multichannel intraluminal impedance (pH-MII) is a combination diagnostic tool used to evaluate the esophageal phase of GER, including pH changes that can diagnose the presence and frequency of gastroesophageal reflux episodes [80]. pH-MII can differentiate reflux events from liquid refluxate with air swallowing events and evaluate ongoing GER symptoms refractory to therapy and the efficacy of anti-acid therapy [69,81]. A liquid gastric emptying nuclear medicine scan (“Milk Scan”) using a milk-based formula labelled with Tc-99m sulfur colloid is also helpful to understand gastric motility in these infants, showing a correlation between GER and delayed gastric emptying [82]. 

Pharyngo-esophageal high-resolution manometry (PE-HRM) is an advanced tool used to understand abnormal aerodigestive motility reflexes and help guide management. In a cohort of 74 preterm infants with GER symptoms evaluated by both pH impedance and provocative esophageal manometry, a higher correlation of GER symptoms was noted with increasing stimulus volumes used for esophageal provocation, highlighting the role of esophageal peristaltic reflexes [83]. In another study, both VFSS and PE-HRM were performed in infants presenting with feeding difficulties; the infants in this cohort were divided into a study group and control group based on the presence or absence of penetration/aspiration on the VFSS, respectively. In infants with penetration/aspiration risk, PE-HRM showed significant abnormalities in pharyngo-esophageal peristalsis reflexes, including increased deglutition apnea time, increased symptoms with pharyngeal stimulation, and decreased distal esophageal contractility with barium swallows [84]. The North American Society for Pediatric Gastroenterology, Hepatology and Nutrition (NASPGHAN) recommends manometry testing when a motility disorder is suspected in children presenting with reflux symptoms [69].

### 4.4. Pharmacological Interventions

Pharmacological interventions are often the last resort in the neonatal period and are frequently limited to treating GER symptoms. The role of anti-acids is limited and recommended to treat GER only after optimizing non-pharmacological measures, including thickening feeds, modifying feeding volume and frequency, and positional therapy [85]. The American Academy of Pediatrics Committee of Fetus and Newborn clinical report on GER recommends using pharmacological interventions sparingly in neonates due to limited evidence and risk of potential harm with gastric acid blockade [85]. However, in older infants, GER tends to be a significant concern as the immaturity-related sucking–swallowing incoordination improves. The pediatric GER clinical practice guidelines from the NASPGHAN recommend using a 4–8-week course of either histamine receptor antagonists (H2RA) such as Famotidine or proton pump inhibitors such as Omeprazole or Lansoprazole (if H2RAs are unavailable or contraindicated) to treat reflux-related erosive esophagitis in infants and do not recommend their use in treating crying, distress, or visible regurgitation in otherwise healthy infants [75].

Motility agents such as erythromycin have been used in severe cases of feeding intolerance with dysmotility. Erythromycin is not recommended in preterm infants <32 weeks [86], while its role >32 weeks for feeding intolerance is debated with mixed results in previous studies [87,88]. Additionally, the adverse effects of erythromycin include alteration of the gut microbiome, antibiotic resistance, and infantile hypertrophic pyloric stenosis [89]. Overall, given the limited evidence and adverse effect profile, the benefits and risks of using erythromycin should be carefully weighed and discussed with parents or caregivers before using erythromycin. Cyproheptadine, a histamine H1 and serotonin 5HT2 antagonist is another medication used in refractory feeding problems in children as it increases appetite, weight, and height; acceptance to more food varieties; and improved self-feeding [90,91]. The use of these medications is reserved for infants with severe aversion and failure to thrive. 

## 5. Future Directions

Feeding problems encompass diverse interlinked etiologies often grouped together. There is an urgent need to subcategorize feeding problems based on underlying etiology to provide targeted diagnostic and therapeutic support. The diagnosis of dysphagia is limited by available techniques and expertise, and, when diagnosed, there are few reliable treatment options in this delicate population. Parents and caregivers are often frustrated with a lack of a quick fix for feeding problems in infancy. Nevertheless, with patience and perseverance, many interventions show results in due time with delayed gratification. A multidisciplinary team approach to feeding in the NICU with established algorithms for early oral motor stimulation, initiation, and advancement of oral feeds using quality improvement methodologies is vital for the mitigation of future feeding problems [92]. Furthermore, predicting feeding problems in infants based on risk factors, instrumental assessments, and novel technologies such as salivary transcriptomics [19] can potentially target interventions before feeding problems become overtly apparent. Research in this area with large-scale, randomized control trials is highly warranted.

Additionally, as more preterm infants survive into adulthood, the long-term effects and healthcare costs of feeding problems are also expected to increase tremendously in the coming decades [4]. Standardizing definitions for feeding problems is vital to understanding the future prevalence of feeding problems in adults born preterm. Management strategies should consider equitable access to investigations, treatments, services following hospital discharge, and continued care of infants and children with feeding problems, as this is a particular concern for lower socioeconomic status groups [2]. More research is warranted to study the impact of social determinants such as race, ethnicity, and socioeconomic status on feeding problems in adults born preterm.
